# Precision Betacarotene Supplementation Enhanced Ovarian Function and the LH Release Pattern in Yearling Crossbred Anestrous Goats

**DOI:** 10.3390/ani10040659

**Published:** 2020-04-10

**Authors:** Noé M. Lopez-Flores, César A. Meza-Herrera, Carlos Perez-Marin, Dominique Blache, Gerardo Arellano-Rodríguez, Santiago Zuñiga-Garcia, Cayetano Navarrete-Molina, Cristina García De la Peña, Cesar A. Rosales-Nieto, Francisco G. Veliz-Deras

**Affiliations:** 1 Regional Universitary Unit on Arid Lands-URUZA, Chapingo Autonomous University, Bermejillo, Durango 35230, Mexico; 2 Institute for Graduate Studies-IDEP, University of Cordoba, 14014 Córdoba, Spain; 3 School of Agriculture and Environment, The University of Western Australia, Crawley 6009, Australia; 4 Autonomous Agrarian University Antonio Narro, Campus Laguna, Torreón, Coahuila 27054, Mexico; 5 Juarez University of the State of Durango, Gómez Palacio, Durango 35010, Mexico; 6 Agronomy and Veterinary Faculty, San Luis Potosí Autonomous University, San Luis Potosí 78321, Mexico

**Keywords:** goats, beta-carotene, targeted supplementation, reproductive efficiency

## Abstract

**Simple Summary:**

The potential out-of-season supplementation effect of beta-carotene (BETA) upon both ovarian function and the luteinizing hormone LH release pattern in anestrous yearling goats was evaluated. Oral BETA-supply to goats with an enlarged gene-pool of dairy goat breeds, influenced in a positive fashion, the out-of-season ovarian outcomes escorted by changes in the release pattern of LH regarding the Control group. Results denote a likely role of BETA not only as modulator ovarian function measured as ovulation rate and antral follicular number but also involved as modifier of the LH-release pattern. The obtained outcomes would be central in the proposal of out-of-season reproductive schemes to enhance the ovarian outcomes in crossbred dairy goats.

**Abstract:**

The possible out-of-season effect of beta-carotene supplementation on ovulation rate (OR), antral follicles (AFN), and total ovarian activity (TOA = OR + AFN) as related to the LH release pattern in yearling anestrous goats was evaluated. In late April, Alpine-Saanen-Nubian x Criollo goats (*n* = 22, 26 N) were randomly allotted to: (1) Beta-carotene (BETA; *n* = 10, orally supplemented with 50 mg/goat/d; 36.4 ± 1.07 kg live weight (LW), 3.5 ± 0.20 units, body condition score (BCS) or (2) Non-supplemented (CONT; *n* = 12, 35.2 ± 1.07 kg LW, 3.4 ± 0.2 units BCS). Upon estrus synchronization, an intensive blood sampling (6 h × 15 min) was accomplished in May for LH quantifications; response variables included (pulsatility-PULSE, time to first pulse-TTFP, amplitude-AMPL, nadir-NAD and area under the curve-AUC). Thereafter, an ultrasonography scanning was completed to assess OR and AFN. The Munro algorithm was used to quantify LH pulsatility; if significant effects of time, treatment or interaction were identified, data were compared across time. Neither LW nor BCS (*p* > 0.05) or even the LH (*p* > 0.05); PULSE (4.1 ± 0.9 pulses/6 h), NAD (0.47 ± 0.13 ng) and AUC (51.7 ± 18.6 units) differed between treatments. Nonetheless, OR (1.57 vs. 0.87 ± 0.18 units) and TOA (3.44 vs. 1.87 ± 0.45 units) escorted by a reduced TTFP (33 vs. 126 ± 31.9 min) and an increased AMPL (0.55 vs. 0.24 ± 0.9 ng), favored to the BETA supplemented group (*p* < 0.05), possibly through a GnRH-LH enhanced pathway and(or) a direct effect at ovarian level. Results are relevant to speed-up the out-of-season reproductive outcomes in goats while may embrace translational applications.

## 1. Introduction

Beta-carotene (BETA) belongs to the family of carotenoids, phytochemical pigments naturally synthesized in fruits, vegetables, plants, algae, and photosynthetic bacteria [1]. In mammals, carotenoids are mainly metabolized at hepatic level; bovine and equids have the uppermost BETA hepatic content, followed by caprine (3.4 µg/g tissue) [2]. Specifically, BETA is a precursor of retinol (Vitamin A), a fat-soluble vitamin, involved in cellular division and differentiation, bone development and reproductive function [1,2]. While BETA-supply has demonstrated to protect both cells and cellular components against oxygen free radicals through its antioxidant function [3], it has been proposed that BETA is able to act independently as vitamin-A precursor [4]. Nonetheless, inconsistent results regarding the effect of BETA-supplementation upon reproductive function have been described, with negative effects [5], positive effects [6] and even no effects [7]. From a reproductive stand point, BETA-supplementation has been associated with increased steroidogenesis in both the corpus luteum and the follicular tissue [6,8,9,10,11].

Besides, the fundamental endocrine actions of the hypophyseal gonadotropins (LH, FSH) and other intra-ovarian molecules acting either in an autocrine or paracrine fashion (i.e., IGF-1 and 2) upon both follicular growth and oocyte maturation, have been also established [12,13,14]. Moreover, the communication among the hypothalamic-pituitary-gonadal (HPG) axis is tightly controlled by an extremely complex arrangement of neuronal inputs ruled not only by photoperiodic, thermoperiodic and nutrigenomic hints but also by the metabolic status of the reproductive animal [15,16]. Furthermore, BETA has shown to down-regulate the estrogen-induced transactivation of the estrogen receptor [17] supporting the possible role of BETA as an HPG-modulating molecule.

The ovary has as primordial task to produce mature oocytes; to accomplish that, an impeccable and well harmonized dialogue among the main ovarian cellular groups (i.e., theca, granulosa and the oocyte) must occur [18,19,20]. Tonic secretion of pituitary LH is the result of a well-coordinated interaction between a brain stimulus and an inhibitory feedback from the gonads; unquestionably, the GnRH pulse generator is the initiator of the LH pulse [20,21]. The GnRH pulse generator has been located within the arcuate nucleus and seems to be composed by a complex array of kisspeptin neurons and their projections merging with GnRH dendrons in the median eminence, suggesting that such complex of arcuate/infundibular kisspeptin neurons are, per se, the GnRH pulse generator in mammals [22]. Interestingly, nutritional supplementation with a mixture of BETA, polyphenolic compounds, and probiotics demonstrated to up-regulate genes involved in both the activation of cellular gonadotropes and up-regulation of GnRH genes [23]. While the involvement nutritional supplementation as a modulator of the HPG axis has been proposed [8,9,10,11,12,13,14,15,16,20,23,24,25], previous studies of our group have demonstrated an interesting role of BETA as a modulator not only of serum insulin [18], triiodothyronine [26], as well as the GH-IGF-1 system [6] but also upon some selected blood metabolites [27]. Building on these previous findings, we hypothesized an out-of-season positive effect of BETA supply on both ovarian function and the releasing pattern of LH across time in yearling goats; this study attempts to solve such inquires.

## 2. Material and Methods

### 2.1. General

All the procedures, methods and managing of the trial units used in this research were done in strict agreement with recognized recommendations for ethical use, care and welfare of animals in research at worldwide [28] and nationwide [29] levels, with institutional approval reference number UACH-DGIP-REBIZA-IBIODEZA/18-086-C-80.

### 2.2. Location, Environmental Conditions, Animals, and Their Management

The present study was carried out at the Chapingo Autonomous University, Regional University Unit on Arid Lands, (UACH-URUZA; 26° N, 103° W; 1117 m). Yearling anestrous Alpine-Saanen-Nubian x Criollo goats (*n* = 22, live weight (LW) = 29.17 ± 1.02 kg, 3.45 ± 1.02 units, body condition score (BCS)) were used under a long-day photoperiodic conditions during April and May (i.e., natural anestrous season at 26° N). In a weekly periodicity, LW and BCS were recorded previous to feeding; BCS was determined on a five-point scale (from 1 = emaciated to 5 = obese) by an experienced technician.

### 2.3. Experimental Design and Treatment Groups

**Figure 1:**
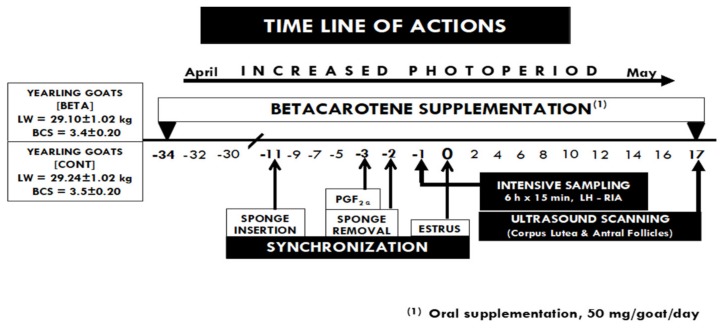
A graphic illustration of the experimental protocol, including the duration of long-term beta-carotene supplementation and estrous synchronization (for more details, see the text). An intensive blood sampling (every 15 min for 6 h) for luteinizing hormone (LH) measurements was performed 36 h prior to the estrus day (day 0). Thereafter, an ultrasonography scanning was performed on day 17 post-estrus to relate the LH secretion pattern and total ovarian activity (TOA = corpus lutea + antral follicles), measured as number of the observed structures present in each ovary on day 17 post-estrus, in yearling crossbred goats (*n* = 22) supplemented with beta-carotene or serving as controls and kept under natural photoperiodic conditions (April–May, 26° N).

In late April, animals were randomly located in individual pens to form two experimental groups: (i). beta-carotene (BETA; *n* = 10, LW = 29.1 ± 1.02 kg, BCS = 3.4 ± 0.2 units) and (ii). Control (CONT; *n* = 12; LW = 29.2 ± 1.07 kg, BCS = 3.5 ± 0.2), with similar LW and BCS between treatment groups. The BETA-goats were orally supplemented with beta-carotene (50 mg/goat/day, mixed with mineral salts) (Syntex-Roche de Mexico; Guadalajara, Jalisco, Mexico) during the entire experimental period, which lasted from 34 d pre- to 17 d, post-estrus. The experimental groups received a basal diet twice per day (0700 and 1600) of alfalfa hay (14% crude protein (CP), 4.7 net energy for maintenance (NEm) MJ/kg), corn silage (8.1% CP, 6.7 NEm MJ/kg), and corn grain (11.2% CP, 9.9 NEm MJ/kg) in a mixed-ration, balanced to cover their net energy requests for maintenance (Table 1) [30]. Both groups had free access to water and shaded areas. Composition values of the components of the basal diet (Dry Matter (DM)% basis) were gotten from representative samples taken thru the experimental period and analyzed based on formerly defined techniques [31]. Allowances of the basal diet and BETA were individually offered to each goat. Because the basal diet was completely consumed by all goats, it is assumed that every experimental unit consumed the same BETA shares from the basal diet. Consequently, the only difference in BETA intake between treatments was the beta-carotene supply provided to the BETA-group. Hence, the effect to offer or not supplemental BETA in both treatment groups was assessed. The central actions executed during the experimental period are depicted in Figure 1.

### 2.4. Estrus Synchronization, Blood Sampling, and LH Quantification

Estrus was synchronized (day 23) with intravaginal sponges containing 45 mg of fluorogestone acetate (Chronogest^®^; Intervet International B.V., Boxmeer, Holland) left in place for 10 days; 9 days after insertion of the sponges (day −3; day 0 = estrus), goats received a single i.m. dose of 1 mL of prostaglandin F_2∝_ analogue (0.075 mg of D-cloprostenol/goat; Prosolvin-C^®^, Intervet International B.V., Boxmeer, Holland). Thereafter, on day −2, sponges were removed and 24 h later (day −1) five goats from each group were randomly selected to undergo an intensive blood sampling. Blood samples (10 mL) were collected every 15 min for 6 h, starting 3 h after the morning feeding, by jugular venipuncture into sterile vacuum tubes (Corvac; Kendall Health Care, St. Louis, MO, USA) and allowed to clot at room temperature for 30 min. Serum was separated by centrifugation (1500× *g*, 15 min), decanted and transferred to polypropylen microtubes (Axygen Scientific, Union City, CA, USA) for storage at −20 °C until hormonal analysis. Peripheral serum LH concentrations were determined in duplicate in a single radioimmunoassay (RIA) as previously described [32]. The value of an intra-assay coefficient of variation (CV) for LH quantification was 10% and the assay detection limit was 0.2 ng/mL; the Munro algorithm was used to detect LH pulses [33]. The LH basal levels were quantified as the average of the lowest points along the sampling period as previously defined [34,35].

### 2.5. Ultrasonographic Scanning of Ovarian Activity

On day 17 post-estrus, thru the end of the luteal phase in middle May, an ultrasonographic scanning was performed to evaluate the ovarian activity, by a qualified operative, using a 7.5-MHz linear-array transducer (Toshiba Medical Systems Ltd., Crawley, UK). Each ovary was scanned and the corpus luteum number (OR) and the antral follicle (AF) number were recorded as previously outlined [36]. Finally, we define to total ovarian activity (TOA) as the sum of AF and OR recorded in each animal within experimental group.

### 2.6. Statistical Analyses

Live weight, body condition score and serum LH concentrations across time were evaluated by split-plot ANOVA for repeated measures in the same animal. Treatments were included in the main plot, and tested using animal within treatment as the error term. The components within the subplot included Time and time × treatment, and were tested by the residual mean square [37]. When significant F values were observed, mean separation was done using the LSMEANS-PDIFF option of the PROC GLM. The ovarian variables and LH-AUC were compared by ANOVA-CRD; due to the non-parametric nature of LH pulse frequency, LH-PULSE was analyzed using the Kruskall-Wallis test. The response variables were evaluated for normality using the Shapiro–Wilk test for normality and transformed by log 10 transformation to overcome skewness in the data for basal and mean LH concentrations and LH pulse amplitude. Where a significant effect of time, treatment or interaction was detected, data were compared across time. The mentioned statistical analyses were solved by means of the GLM procedures of SAS (SAS Inst. Inc. V9.1, Cary, NC, USA). Pearson’s correlations were used to evaluate the associations among LW, BCS and the number of luteal structures. Non-transformed data are presented for ease of interpretation and expressed as least-square means ± standard error (SE); the most conservative SE is presented.

## 3. Results

**Figure 2:**
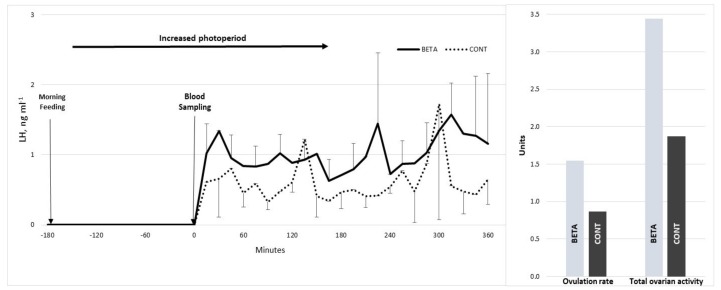
Serum luteinizing hormone concentrations (LH; ng/mL) across time (left panel) and ovulation rate (OR, units), and total ovarian activity (OR + antral follicles, units) (right panel) in crossbred (Alpine–Saanen–Nubian × Criollo; *n* = 22) yearling goats supplemented with beta-carotene (BETA) and non-supplemented (CONT) during the anestrous season (April–May) under semiarid conditions in Northern Mexico (26° N).

Both LW and BCS at the beginning or end of the experiment were 29.17 ± 1.02 kg and 3.4 ± 0.17 or 35.81 ± 1.07 kg and 3.4 ± 0.2, respectively. No differences between groups for both variables occurred either at the beginning (*p* > 0.05) or during the entire (*p* > 0.05) experimental period. Besides, differences (*p* < 0.05) also occurred between treatments concerning the LH release pattern (Figure 2). While no differences for PULSE (4.1 ± 0.9 pulses/6 h), NAD (0.47 ± 0.13 ng) and AUC (51.7 ± 18.6 units) occurred between groups, both a reduced TTFP (33.0 vs. 126.0 ± 31.2 min) as well as an increased AMPL (0.55 vs. 0.24 ± 0.7 ng) favored to the BETA-supplemented goats. Moreover, regarding to the ovarian outcomes, both OR (1.57 vs. 0.87 ± 0.18 units) and TOA (3.44 vs. 1.87 ± 0.45 units), favored to the BETA supplemented group (*p* < 0.05) (Table 2). In addition, positive correlations were detected between OR regarding LW (r^2^ = 0.52; *p* < 0.05), BCS (r^2^ = 0.57, *p* < 0.05), AF (r^2^ = 0.89, *p* = 0.01), OR (r^2^ = 0.65, *p* = 0.03) as well as between AMPL and OR (r^2^ = 0.55, *p* = 0.06).

## 4. Discussion

The observed results of the study support our working hypothesis in that BETA-supply enhanced ovarian function in yearling goats with escalations in ovulation rate, antral follicle population, and total ovarian; such ovarian activity was escorted with changes in the LH-release pattern (time to first LH pulse and LH amplitude). However, no differences regarding LW and BCS, as well as pulsatility, nadir, and area under the curve of LH occurred between experimental groups. Such a physiological scenario suggests that BETA supply may had exerted a direct action at the ovarian level, acting throughout a GnRH-LH dependent pathway, likely acting throughout the “dynamic effect” of BETA supply. Remarkably, as the BETA-supply was constant up to 17 d post-estrus, such BETA-supply which positively affected the antral follicle number, can be demarcated as an “acute effect” of BETA-supplementation upon the growth of the antral follicle population. Consequently, it can be proposed that BETA supply generated a physiologic ovarian setting that promoted an increased follicular steroidogenesis and growth, positively acting upon the ovarian physiology throughout both the dynamic and the acute effect of BETA-supplementation, escorted by specific changes in the LH-release pattern.

In domestic livestock, nutritional supplementation modulates not only the timing and functionality of endocrine systems but also regulates seasonal shifts in reproductive activity, while imposing diverse reproductive outcomes (i.e., ovulation rate and fertility) [12,13,14,15,16,19,20,38,39]. For such reasons, nutritional supplementation is an interesting clean, green, and ethical management alternative with respect to the use of diverse hormonal treatments to enhance reproductive efficiency. To better understand our results, it is required to consider that nutritional supplementation (inputs) affects reproductive responses (outputs) through changes in the metabolic status. The last not only in the long term (“static effect”) but also in the short-to-middle term (“dynamic effect”) through an increased feeding over 3–4 weeks before mating; the heavier the females, the greater the ovulation rates as compared to their lighter counterparts. Moreover, enhanced reproductive outcomes can be also obtained by supplying a nutritional boost in a very short period of time (i.e., less than 5 to 7 days), without any perceptible effect upon live weight; the last has been referred to as “immediate nutrient effect”, “acute effect”, or “focus supplementation” [13,19,20,39,40,41]. Diverse studies have proposed that the stimulating effects of nutritional supplementation on follicular development can be linked to the effect of such increased nutritional supply not only upon LH concentrations but also with respect to the LH release pattern, which may directly act upon the ovarian tissue. Certainly, malnourished animals reach puberty at later stages or chronological age due to a decrement in GnRH pulsatility, which in turn leads to corresponding decreases in LH pulses [42]. Furthermore, changes in specific nutritional compounds and different biomolecules, such as BETA, may support increases not only in follicular growth in an FSH-dependent process but also upon increases in the LH-release pattern and then in ovulation rate [12,13,14,15,16,18,19,20,43]; the last physiological and neuroendocrine scenario possibly occurred in the BETA-group. The effect of nutritional supplementation on reproduction is clearly complex. The static effect has been associated with the body condition status (i.e., adiposity); while increases in body condition upsurges adiposity, such an augment in energy-reserves increases, in turn, follicular growth and ovulation rate. Besides to the previously mentioned FSH and LH involvement, the systemic and/or intra-follicular actions of the insulin-glucose and the IGF-1-leptin systems, should be also involved [12,13,20,39].

Going back to our results, the observed differences in the LH release pattern and the increased ovarian activity observed in the BETA group, suggest not only the activation of the GnRH-LH pathway but also a direct BETA effect upon ovarian tissue. Moreover, a potential positive role of BETA-supply along the aromatization progression in the antral follicles population can be suggested; a steroidogenic setting formerly proposed [24]. When evaluating the BETA uptake by ovarian and uterine tissues and its possible influence upon steroidogenesis during the estrous cycle, not only an increased plasma concentration of estradiol occurred between days 0 to 4 post-ovulation but also an augmented total uterine protein concentration favored to the BETA supplemented females [44]. Such findings point out to BETA as an enhancer not only of the production of healthier follicles and oocytes but also in the concentrations of estradiol, progesterone and uterine proteins, providing an ideal ovarian performance as well as an improved uterine milieu prone to an enhanced embryo implantation and development.

In addition, increases of intra-ovarian reactive oxygen species (ROS) are aligned to increases in maternal age [45]. Such a physiological-chronological scenario highlights the key role of BETA not only as a very effective quencher of ROS, but also as an important molecule which enhances both ovarian development and steroidogenesis. In an elegant study, the possible effect of BETA on oocyte maturation under oxidative stress and the involved underlying mechanisms was explored [46]; while ROS inhibited oocyte development/maturation and parthenogenetic activation, BETA actions vanished such deleterious mechanisms, enhancing ovarian activation and development. Additionally, the protective BETA effects against the oxidative damage in ovarian tissue facing ischemia throughout different expression patterns of the antioxidant enzymes superoxidase dismutase (SOD) and glutathione (GSH), plus the malondialdehyde (MDA) level, which is the end result of lipid peroxidation, have been proposed [47]. Moreover, BETA supply not only restored actin expression, cortical granule-free domain formation, mitochondria homogeneous distribution and nuclear maturation [47] but also reduced both ROS formation and cell apoptosis [1]; such results point out to BETA as an improver of both ovarian function and oocyte quality. Unquestionably, the specific site of action of BETA-supply thru the HPG axis will not be recognized without additional enquiries. In this respect, it has been proposed that serum vitamin concentrations affect steroidogenesis even after adjustment for oxidative stress, the last throughout an intricate association among beta-carotene-like antioxidants and diverse endogenous metabolic hormones [48].

## 5. Conclusions

Our main research outcomes establish that beta-carotene supply enhanced out-of-season follicular development and ovulation rate, escorted by changes in the LH-release pattern in yearling anestrous goats, with non-differences regarding live weight and body condition score. Whether the effects of BETA-supplementation were exerted by differences in the LH-release pattern, or directly exerted upon the ovarian physiology or even mediated through an up-regulation of the ovarian LH-receptors remains as a pending research assignment. Supplementary research designed to expounding the precise endocrine or metabolic effects of BETA along the HPG axis would contribute to a better understating regarding the positive out-of-season ovarian outcomes observed in the yearling BETA-supplemented anestrous goats, notably with a high degree of genes from very seasonal reproduction breeds. The observed research outcomes from our study should aid to speed-up the ovarian responses in anestrous goats while may also embrace interesting translational applications aiming to suppress metabolic-physiologic dysfunctions in aging human oocytes.

## Figures and Tables

**Table 1:** Chemical composition of alfalfa hay, corn silage and corn grain samples which conformed the basal diet of yearling crossbred goats (*n* = 22) under natural photoperiodic (April–May, 26° N) ^a^.

Item	Alfalfa Hay	Corn Silage	Corn Grain
Nutrient composition ^b^	(%)	(%)	(%)
Dry matter ^c^	92.0	35.8	85.3
Crude protein ^c^	15. 8	8.5	9.5
Neutral detergent fiber ^c^	59.9	40.6	9.9
Acid detergent fiber ^c^	42.1	25.0	4.0

^a^ Mineral block offered ad libitum contained (%, weight/weight): NaCl 95; Fe 0.2; Cu 0.033; I 0.007; Zn 0.005; Co 0.0025, ^b^ Composition values (% of diet Dry Matter basis) represent values from five samples taken throughout the experimental period. Samples dried in a forced air stove at 60 °C until constant weight, ^c^ Determined according to the procedures outlined by AOAC, 1990 [31].

**Table 2:** Least square means regarding live weight and body condition score at the onset of treatments (live weight (LW)-initial, kg and body condition score (BCS)-initial, units) and at the ultrasound scanning (LW-ultrasound, kg and BCS-ultrasound, units), ovulation rate (OR, units), total ovarian activity (TOA = OR + antral follicles, AF), and luteinizing hormone (LH) profile across time (pulsatility, time to first pulse, amplitude, nadir, and area under the curve) in cross-bred (Alpine–Saanen–Nubian × Criollo; *n* = 22) yearling goats supplemented with betacarotene (BETA) and non-supplemented (CONT) during the anestrous season (April–May) under semiarid conditions in Northern Mexico (26° N) **^(1)^**.

Variables	BETA	CONT	S.E. ^(2)^
Live weight-initial, kg	29.10 ^a^	29.24 ^a^	1.02
Body condition score-initial, units	3.4 ^a^	3.5 ^a^	0.17
Live weight-ultrasound, kg	36.42 ^a^	35.20 ^a^	1.07
Body condition score-ultrasound, units	3.5 ^a^	3.4 ^a^	0.20
Ovulation rate, units	**1.55 ^a^**	**0.87 ^b^**	0.18
Total ovarian activity, OR + AF, units	**3.44 ^a^**	**1.87 ^b^**	0.45
LH pulsatility, pulses/6 h, units	4.61 ^a^	4.02^a^	0.79
LH time to first pulse, min	**33.0 ^a^**	**126.0 ^b^**	31.28
LH nadir, ng	1.18 ^a^	1.30 ^a^	0.39
LH amplitude, ng	**0.55 ^a^**	**0.24 ^b^**	0.07
LH area under the curve, arbitrary units	36.5 ^a^	66.9 ^a^	4.47

^(1)^ Yearling goats were weighed and body conditioned at the onset of the experimental period (early April) and at the ultrasonographic scanning (middle May). Goats confronted an increased natural photoperiod (April–May; anestrous season). While no differences among treatments occurred regarding neither LW nor BCS at either stage of the experimental period, differences were observed regarding the LH release pattern as well as OR and TOA, favoring to the BETA supplemented group (*p* < 0.05). ^(2)^ Most conservative standard error is presented. ^a, b^ Different superscripts within variable, show differences (*p* < 0.05).
